# AD or Non-AD: A Deep Learning Approach to Detect Advertisements from Magazines

**DOI:** 10.3390/e20120982

**Published:** 2018-12-17

**Authors:** Khaled Almgren, Murali Krishnan, Fatima Aljanobi, Jeongkyu Lee

**Affiliations:** 1 College of Computing and Informatics, Saudi Electronic University, Riyadh 11673, Saudi Arabia; 2 College of Engineering, University of Bridgeport, Bridgeport, CT 06614, USA

**Keywords:** deep learning, convolutional neural network, image recognition, advertisement detection, advertisement detection

## Abstract

The processing and analyzing of multimedia data has become a popular research topic due to the evolution of deep learning. Deep learning has played an important role in addressing many challenging problems, such as computer vision, image recognition, and image detection, which can be useful in many real-world applications. In this study, we analyzed visual features of images to detect advertising images from scanned images of various magazines. The aim is to identify key features of advertising images and to apply them to real-world application. The proposed work will eventually help improve marketing strategies, which requires the classification of advertising images from magazines. We employed convolutional neural networks to classify scanned images as either advertisements or non-advertisements (i.e., articles). The results show that the proposed approach outperforms other classifiers and the related work in terms of accuracy.

## 1. Introduction

Nowadays, digital marketing has gained a high level of attention due to the massive use of the internet. Many Internet sites, including Facebook, Google, and Instagram, attract billions of users daily [1]. This phenomenon has driven many companies to use the Internet for marketing; they post their advertisements on online sites, similar to how advertisements are placed on the pages of magazines.

Companies tend to keep their marketing budget confidential for competition purposes; however, rival companies are interested in finding out the marketing budget of their competitors to improve their own marketing strategies. Therefore, they are estimating their competitors’ marketing budgets by identifying their advertisements manually; however, this process takes time and effort due to the fact that the number of advertisements has increased tremendously. In addition, this makes it almost impossible to process the images manually. Therefore, an efficient computerized method is needed to detect advertisements in a reasonable timeframe.

In this paper, we propose an approach to detect advertisement images from magazines based solely on the visual content. This approach was tested on a large dataset which contains images that were scanned from several magazines. We took advantage of the outstanding performance of deep learning in solving problems in multiple fields, such as image processing [2,3], object detection and recognition [4], healthcare [5], multi-threading [6], drug discovery [7], causal shape transformation [8], handwritten digit recognition [9], segmentation [10], optimization [11], texture classification [12], and sentence classification [13,14]. Therefore, we employed a convolutional neural network for extracting features from images to classify the scanned images into advertisements and non-advertisements. The summary of our contribution is presented below:

1. The image dataset is a collection from various magazines, and it contains both advertisement and non-advertisement images in a variety of designs.
2. We optimized the convolutional neural networks using different filters to classify images as advertisements or non-advertisements from magazines.
3. A comparison was made with other classifiers, and it shows that our approach is feasible for detecting advertisements from a large dataset and outperforms other classifiers.

The remainder of the paper is organized as follows. Related works are presented in Section 2. In Section 3, we present our approach. In Section 4 and Section 5, we discuss our experimental setup and results, respectively. Conclusions and future work are provided Section 6.

## 2. Related Work

The previous works discussed here have used text and visual features to detect advertisements. Ouji et al., used K-NN and Adaboost to classify advertisements from the newspaper [15]; they used visual features, such as colored text, colored background, multiple colors, photo areas, and irregular alignments. Chu and Chang proposed a framework to detect and segment advertisements from newspaper or website snapshots using visual features and semantic features embedded in advertisements [16]. They categorized advertisements into a set of categories, such as manufacturing, wholesale, real estate, and education, and they used a convolutional neural network to extract visual features and then used an SVM to perform advertisement classification. They extracted semantic concepts from images using the VIREO374 package to categorize advertisements [17]. Peleato et al. classified advertisements into four classes (real estate, vehicles, employment, or other) based on text information using Naive Bayes [18]. The previous research works have used visual and text features from images to detect advertisements. However, in this study, we focus only on the visual content to classify images from magazines.

## 3. Approach

**Figure 1:**
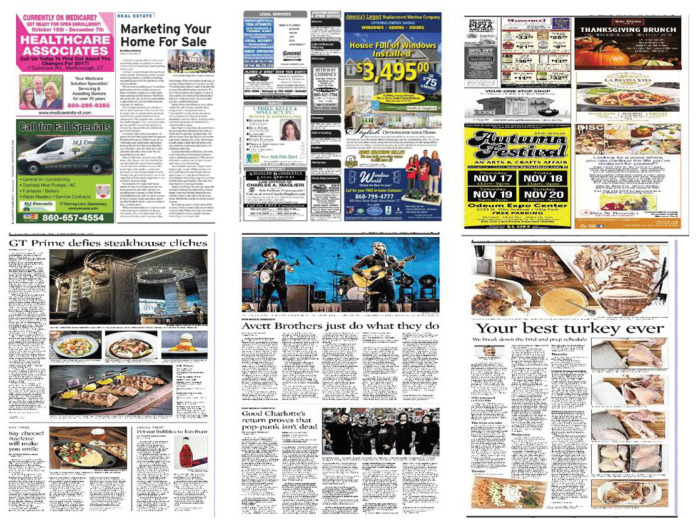
Sample advertisement and non-advertisement images from different magazines. The top three images are advertisement images, while the rest are non-advertisement images.

**Figure 2:**
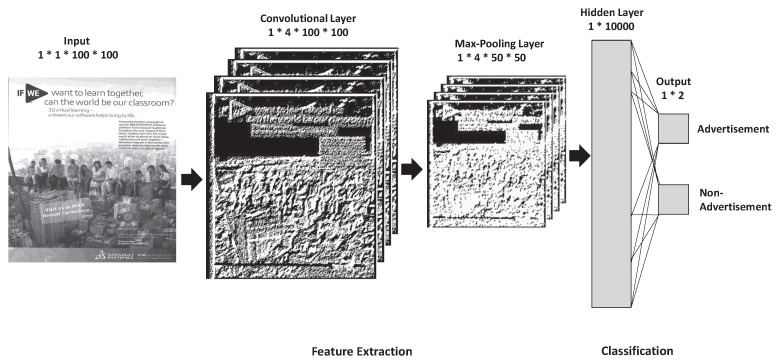
The convolutional neural network architecture.

In this study, we employed convolutional neural networks to detect advertisements within scanned images from magazines. Figure 1 shows several sample images of both advertisements and non-advertisements. As you can see, there are no clear visual features to classify them, e.g., colors or shape. In order to address this, the proposed architecture consists of three layers: the convolutional layer, max-pooling layer, and fully connected layer (hidden and output layers). The architecture is illustrated in Figure 2. Our approach follows two steps:

1. Feature extraction, and
2. Classification.

### 3.1. Feature Extraction Layer

The feature extraction layer consists of the convolutional layer with ReLU activation and the max-pooling layer. The input for the convolutional layer is a batch of images. The images are 100 × 100 pixels, which are grayscale. We used four filters for the convolution process, which are basic edge detectors. The convolutional layer is a basic edge detector that aims to extract low-level features (edges), where the edges are obtained by convolving the input with its respective filter to learn feature representation [19]. The following equation is used to implement the convolutional layer.

(1) $$y_{i}^{l}=f\left(\sum_{i}x_{i}^{l}*k_{ij}^{l}+b_{j}^{l}\right)$$

where $k_{ij}^{l}$ is the convolutional filter, $x_{i}^{l}$ is the input image, $b_{j}^{l}$ is the bias, and * is the convolution operation. We used ReLU activation to remove the negative pixels from the output of the convolution process. This function is also used for fast training [20,21]. The ReLU function is shown below:

(2) z=max(0,y)

where *y* is the output from the convolutional process.

The pooling layer is used to reduce the size of the features extracted from the convolutional layer to control overfitting and downsampling [22]. There are many pooling processes available, and, in this study, we used max-pooling with a window size of 2 × 2. Max-pooling functions by:

1. Reducing the number of parameters within the model. This is called downsampling (or subsampling), and it retains the most important feature, thus reducing the computational cost.
2. Generalizing the results from a convolutional filter. This makes the detection of features invariant to scale or orientation changes.

Also, max-pooling provides better performance than other pooling processes because it converges faster [23]. The following equation illustrates the max-pooling process:

(3) h=max(z)

where *z* is the output from the convolutional layer.

### 3.2. Classification Layer

**Figure 3:**
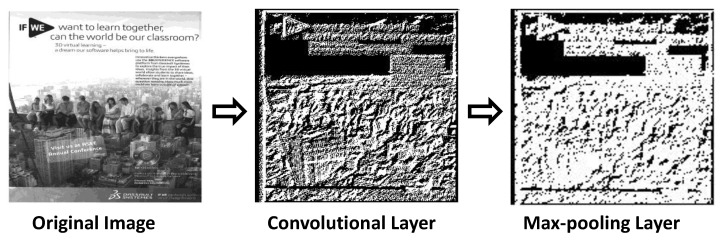
The output from the process for detecting advertisements from magazines.

The classification layer is a fully connected layer. The main purpose of this layer is to classify images as advertisements or non-advertisements. The fully connected layer tries to learn global feature representation [24] for both advertisement and non-advertisement images. The output from the max-pooling layer is fed into the hidden layer, which contains 10,000 neurons. The hidden layer multiplies each input by the weight, as shown in the following equation:

(4) a=f(wh+b)

where *w* is the weight (the weights are initialized using random numbers), *b* is the bias, and *h* is the max-pooling output. Then, we applied the sigmoid activation function to normalize *a* [25]. The parameters of the convolutional neural network architecture are shown in Table 1. An example of the output is shown in Figure 3.

As for the objective function, we used cross-entropy, which computes the sigmoid cross-entropy between the predicted and actual class. It is computed as follows:

(5) $$Cross_{E}ntropy=targets*-log\left(sigmoid\left(logits\right)\right)+\left(1-targets\right)*-log\left(1-sigmoid\left(logits\right)\right)$$

where targets refers to the actual class, and logits refers to the predicted class.

## 4. Experimental Setting

### 4.1. Dataset

For the experiment, we scanned 3885 images from multiple magazines, such as Departures, Mechanical Engineering, Appliance Design, and ASEE magazines. The dataset contains 2078 advertisement images and 1807 non-advertisement images. We split the dataset randomly into two groups (85% and 15%), the training and testing datasets, in order to train and test our model. The training set contains 3332 images while the testing set contains 553 images. The training dataset contains 1914 advertisement images and 1418 non-advertisement images while the testing dataset contains 164 advertisement images and 389 non-advertisement images. The dataset characteristics are summarized in Table 2. The images collected from magazines come in a variety of sizes. Therefore, all the images were resized to 100 pixels in width and 100 pixels in height. Also, the scanned images were converted to grayscale images. The images were resized and converted to grayscale in order to reduce the computational cost when training our architecture; thus, the image information is kept intact.

### 4.2. Filters

**Figure 4:**
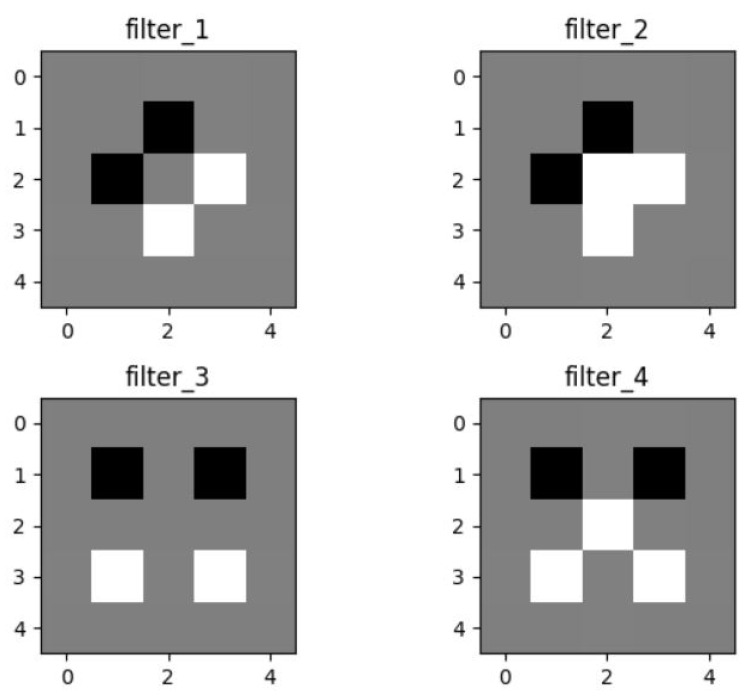
Edge detection filters.

We used four filters in the convolution layer for detecting the low-level features, which, in this case, are the edges. Four filters with a size of 5 × 5 were created without depth because the input image is in grayscale and has no depth. One filter detects horizontal edges, one detects vertical edges, and the remaining two filters detect diagonal edges in the input image during the convolution process. Figure 4 shows the edge detection filters used in our model for the convolution process. The filters in the diagram are the basic edge detector filters. These filters detect the horizontal and vertical edges.

### 4.3. Implementation

The steps involved in the implementation are as follows:

1. Convolution Layer: Convolving each filter with the input image.
2. ReLU Layer: Applying ReLU activation on the feature maps.
3. Max-Pooling Layer: Applying the pooling operation to the output of the ReLU layer.
4. Fully Connected Layer: A neural network with one hidden layer to perform the classification.

In order to implement the convolutional neural network, a naive approach using Numpy for performing feed-forward and back-propagation processes was adopted. We used one convolutional layer with four filters for extracting low-level features, which are edges, along with ReLU activation to remove negative pixels, one max-pooling layer to capture the maximum change in the pixels, and a fully connected layer which has one hidden layer that performs the classification. For comparing our model with other classifiers, we used Keras [26]. We used Keras to compare our model with the other classifiers. Keras is a widely used high-level neural network API, which is written in Python. The other classifiers were implemented using Scikit-learn [27].

### 4.4. Mini-Batch Gradient Descent

We used the mini-batch gradient descent algorithm for training our network. Gradient descent is an optimization algorithm often used for finding the weights or coefficients of machine learning algorithms, such as artificial neural networks and logistic regression. The model makes predictions on training data and uses the error on the predictions to update the model in order to reduce the error. The model’s update frequency is higher than the batch gradient descent, which allows for a more robust convergence, avoiding local minima. The batched updates result in a computationally more efficient process than stochastic gradient descent. Batching enhances efficiency by not having all the training data in both memory and algorithm implementations [28].

### 4.5. Evaluation

In order to evaluate our proposed approach and compare between the different approaches, we generated a confusion matrix to calculate the overall accuracy, sensitivity, and specificity of the models. The overall accuracy is computed as follows:

(6) $$Accuracy=\frac{TP+TN}{Total\;Number\;of\;Samples}$$

where TP refers to advertisements images that are correctly identified as an advertisement, and TN refers to non-advertisement images that are correctly identified as non-advertisements. The sensitivity and specificity are used to evaluate the ability of the models to predict advertisements and non-advertisements. They are computed as follows:

(7) $$Sensitivity=\frac{TP}{TP+FN}$$

(8) $$Specificity=\frac{TN}{TN+FP}$$

where FN refers to advertisement images that were identified as non-advertisement images, while FP refers to non-advertisement images that were identified as advertisement images. In order to validate our results, we performed *k*-fold cross-validation, where k=4.

## 5. Results

As shown in Table 3, our proposed approach outperforms all other approaches in terms of overall accuracy. This shows that the proposed approach is capable of classifying images into advertisements and non-advertisements. It achieves an accuracy of 78%. However, for classifying advertisements, it achieves a sensitivity of 57%, and, for classifying non-advertisements, it achieves a specificity of 87%. This shows that our approach performs better in predicting non-advertisement images.

**Figure 5:**
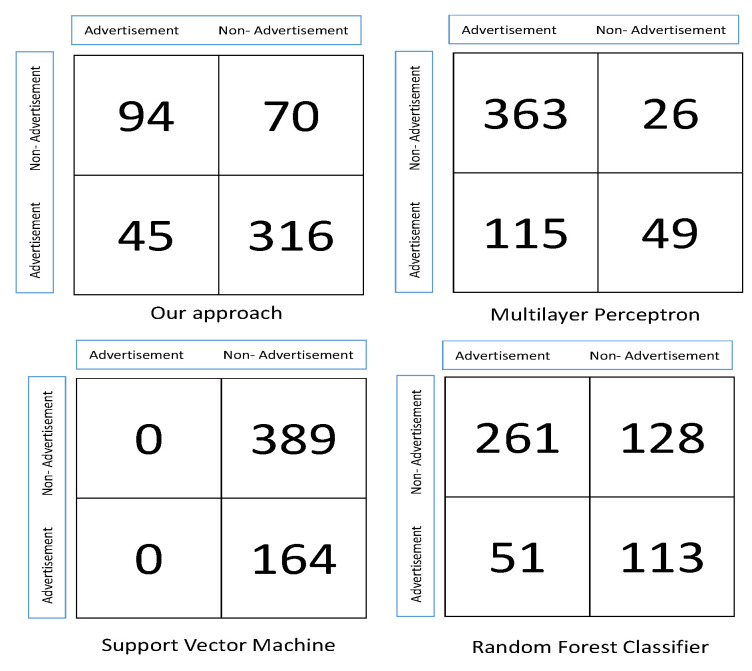
Confusion matrices for each approach.

Table 4 and Table 5 compare the sensitivity and specificity of our model with those of the other algorithms. Confusion matrices for all the algorithms are shown in Figure 5.

## 6. Conclusions and Future Work

In this research, we detected advertisements from multiple magazines. We employed a convolutional neural network to extract meaningful features from the scanned images. We tested our approach on a large dataset, which contains 3885 images from different magazines. The results show that our approach is feasible for detecting advertisements, and it also outperforms other classifiers in terms of accuracy. In the future, we will investigate different features of magazine advertisements, such as semantics, and work with different filters. Our future goals include classifying images as advertisements and non-advertisements and then classifying those identified as advertisements even further by source (such as magazine advertisements, newspaper advertisements, etc.). We also aim to determine the content of advertisements and categorize them by industry (such as food, tourism, automobiles, etc.). This would make our model’s output include more than two classes. To achieve this, we will construct the architecture for our convolutional neural network, which includes factors such as the number of convolutional and max-pooling layers, the arrangement of convolutional and max-pooling layers, the number of hidden layers, and the number of neurons in each hidden layer for the fully connected layer.

## Figures and Tables

**Table 1:** Summary of the convolutional neural network.

Layer	Feature Map Size	Kernel Size
Input	1 × 1 × 100 × 100	-
Convolution	1 × 4 × 100 × 100	5 × 5 × 4
Max-pooling	1 × 4 × 50 × 50	2 × 2
Fully connected	1 × 10,000	
Output	1 × 2	

**Table 2:** Dataset.

Dataset\Classes	Advertisements	Non-Advertisements	Total
**Training**	1914	1418	3332
**Testing**	164	389	553
**Total**	2078	1807	3885

**Table 3:** Experiment results.

Algorithm	Accuracy
Our approach	78%
Multilayer Perceptron	74%
Random Forest Classifier	67%
Support Vector Machine	29%

**Table 4:** Sensitivity.

Algorithm	Sensitivity
Our approach	57%
Multilayer Perceptron	93%
Random Forest Classifier	67%
Support Vector Machine	0%

**Table 5:** Specificity.

Algorithm	Specificity
Our approach	87%
Multilayer Perceptron	29%
Random Forest Classifier	68%
Support Vector Machine	100%
